# Lipid-Mediated Insertion of Toll-Like Receptor (TLR) Ligands for Facile Immune Cell Engineering

**DOI:** 10.3389/fimmu.2020.00560

**Published:** 2020-04-22

**Authors:** Michael H. Zhang, Emily M. Slaby, Georgina Stephanie, Chunsong Yu, Darcy M. Watts, Haipeng Liu, Gregory L. Szeto

**Affiliations:** ^1^Chemical, Biochemical, and Environmental Engineering, University of Maryland Baltimore County, Baltimore, MD, United States; ^2^Marlene and Stewart Greenebaum Comprehensive Cancer Center, University of Maryland, Baltimore, MD, United States; ^3^Department of Chemical Engineering and Materials Science, Wayne State University, Detroit, MI, United States; ^4^Center for Biomedical Engineering and Technology, University of Maryland School of Medicine, Baltimore, MD, United States; ^5^Translational Center for Age-Related Disease and Disparities, University of Maryland Baltimore County, Baltimore, MD, United States

**Keywords:** membrane insertion, drug delivery, immunotherapy, adjuvants, Toll-like receptors, cell engineering, T cells, B cells

## Abstract

Cell-based immunotherapies have tremendous potential to treat many diseases, such as activating immunity in cancer or suppressing it in autoimmune diseases. Most cell-based cancer immunotherapies in the clinic provide adjuvant signals through genetic engineering to enhance T cell functions. However, genetically encoded signals have minimal control over dosing and persist for the life of a cell lineage. These properties make it difficult to balance increasing therapeutic efficacy with reducing toxicities. Here, we demonstrated the potential of phospholipid-coupled ligands as a non-genetic system for immune cell engineering. This system provides simple, controlled, non-genetic adjuvant delivery to immune cells via lipid-mediated insertion into plasma membranes. Lipid-mediated insertion (termed depoting) successfully delivered Toll-like receptor (TLR) ligands intracellularly and onto cell surfaces of diverse immune cells. These ligands depoted into immune cells in a dose-controlled fashion and did not compete during multiplex pairwise loading. Immune cell activation could be enhanced by autocrine and paracrine mechanisms depending on the biology of the TLR ligand tested. Depoted ligands functionally persisted on plasma membranes for up to 4 days in naïve and activated T cells, enhancing their activation, proliferation, and skewing cytokine secretion. Our data showed that depoted ligands provided a persistent yet non-permanent adjuvant signal to immune cells that may minimize the intensity and duration of toxicities compared to permanent genetic delivery. Altogether, these findings demonstrate potential for lipid-mediated depoting as a universal cell engineering approach with unique, complementary advantages to other cell engineering methods.

## Introduction

Advances in drug delivery have enhanced our understanding of basic biology and generated novel therapies. In cell-based immunotherapy, drugs are administered systemically to target immune cells *in vivo*, or carried by *ex vivo*-primed autologous immune cells that are reinfused into patients. Strategies to engineer cells as drug-carriers *ex vivo* include genome editing, conjugating biomaterial-based carriers to cell surfaces, and binding of the plasma membrane for passive diffusion of immunostimulatory ligands (1, 2). Despite successful implementation in the clinic, cell-based immunotherapies still face challenges of implementing simple and efficient delivery that improve therapeutic responses. For example, genome editing can decrease cell viability and has low efficiency in some cell types of interest, particularly T cells (3–5). Biomaterial-based drug carriers with defined size, shape, cargo loading, composition, and physiochemical parameters can enable controlled delivery to intracellular cell compartments for autocrine signaling or to surrounding cell surface receptors for paracrine signaling (2). However, optimization of nanoparticle design parameters is complex, and often requires empirical testing due to the variability associated with formulations for each drug-particle combination, causing inconsistent cell loading or uptake (6). Here, we propose a simplified delivery platform that couples diverse biomolecular cargo to phospholipids that directly insert into plasma membranes for universal loading into plasma membranes of cells without the need for gene editing or complex biomaterial design.

Biomolecules have been previously conjugated to phospholipids for cell loading by plasma membrane interactions. Our previous study generated potent vaccines by exploiting the affinity of lipids to bind albumin and deliver antigen and adjuvant conjugates to lymphoid organs (7). *Ex vivo* modification with lipid conjugated immunostimulatory ligands has also been investigated for some adjuvants. In cancer, lipid-mediated delivery of T-cell adjuvants elicit tumor regression in multiple preclinical models of cancer (8, 9). Lipid conjugated adjuvants also strongly associate with plasma membranes of dendritic cells and tumor cells (10, 11). However, previous studies have not fully characterized the efficiency of lipid-mediated insertion into plasma membranes, or expanded this delivery approach to diverse immunostimulatory cargoes.

Toll-like receptors (TLRs) are one family of molecules that are heavily used to enhance immune responses. TLRs are a major contributor to innate immune sensing, and also directly implicated in adaptive immunity during many diseases (12–15). For example, TLR2 is a cell surface receptor that senses molecules from microbial cell-walls. TLR2 activation results in increased pro-inflammatory cytokine secretion, suppression of regulatory T cells, and enhanced sensitivity of cytotoxic T cells (3, 13, 16). TLR9 is an intracellular receptor that senses unmethylated CpG DNA from viruses and bacteria. TLR9 ligands are frequently used as vaccine adjuvants (17, 18), and can directly enhance proliferation and survival of T cells (19, 20). Successful delivery of both TLR2 and TLR9 ligands has demonstrated promising therapeutic responses, particularly in cancer (13, 21–23). However, improved delivery approaches are needed to more easily deliver cell surface and intracellular ligands to diverse immune cells.

We report that lipid-conjugated TLR2 and TLR9 ligands can rapidly and simply insert into immune cell plasma membranes (hereafter termed depoting), delivering ligands to both cell surface and intracellular receptors. We analyzed ligand loading, dynamics of ligand persistence, and activation of mouse immune cells to demonstrate the feasibility of depoting to provide paracrine and autocrine signals that can enhance immune cell function. This study provides proof-of-concept that depoting immunostimulatory ligands into plasma membranes can enhance cell function, highlights key features of this platform including dynamics of single and multiplex ligand loading and turnover, and provides a new method for engineering cell-based therapies that can complement existing methods.

## Methods

### TLR2 and TLR9 Ligands

Synthetic ligands for TLR2, Pam2CSK4 and Pam3CSK4, and their biotinylated variants were purchased from Tocris Bioscience and InvivoGen. TLR9 ligand, CpG oligonucleotide 1826 (5′-tccatgacgttcctgacgtt-3′ with a phosphorothioated backbone) (CpG) and fluorescein (FAM)-labeled CpG, were commercially synthesized (Integrated DNA Technologies). Diacyl stearoyl (C18) lipid conjugated CpG (lipid-CpG) and FAM-labeled lipid-CpG were made as previously described by synthesizing diacyl C18 lipid phosphoramidite and conjugating to either CpG or CpG-FAM on a ABI 394 synthesizer on 1.0 micromole scale (11). Lipid-CpG was purified by reverse phase HPLC with a C4 column (BioBasic4, 200 mm × 4.6 mm, Thermo Scientific). A gradient eluent (Sigma-Aldrich) was implemented with 100 mM triethylamine-acetic acid buffer (pH 7.5) and acetonitrile (0–30 min, 10–100%).

### Isolation of Naïve and Primed Mouse Immune Cells

All procedures with animals and animal-derived materials were approved by the UMBC Institutional Animal Care and Use Committee (OLAW Animal Welfare Assurance D16-00462). C57BL/6 mice and CD45.1^+^ (B6.SJL-Ptprca Pepcb/Boy) congenic mice from Jackson Laboratory were bred in the UMBC animal facility and used for all experiments. Spleens from 12–52 week old C57BL/6 mice were mashed through a 40-μm cell strainer treated with ACK lysis buffer (1 mL per spleen, ThermoFisher) for 5 min at 25°C to lyse red blood cells. Naïve CD4^+^ and CD8^+^ T cells were isolated using a negative selection cocktail containing the following biotinylated mouse antibodies (Biolegend): TCR γ/δ (clone GL3), CD24 (clone M1/69), TER-119 (clone TER-119), CD49b (clone HMα2), CD45R/B220 (clone RA3-6B2), CD19 (clone 6D5), CD11c (clone N418), and CD11b (clone M1/70). B cells were isolated from splenocytes using a negative selection cocktail containing the following biotinylated mouse antibodies: CD43 (clone 1B11), CD90.2 (clone 30-H12), Gr-1 (clone RB6-8C5), TER-119 (clone TER-119), CD49b (clone HMα2), CD11b (clone M1/70), CD8 (clone 53-6.7), and CD4 (clone H129.19). Antibody-bound cells were depleted with Rapidsphere streptavidin magnetic beads according to the manufacturer's instructions (STEMCELL Technologies). Cells with >90% purity were used for experiments.

Primed T cells were obtained by culturing splenocytes in complete RPMI 1640 media supplemented with 10% fetal bovine serum (FBS; ThermoFisher), Concanavalin A (2 μg/mL; Sigma-Aldrich), and IL-7 (2 ng/mL, Biolegend) at 37°C for 2 days. Ficoll-Paque Plus (GE Healthcare Life Sciences) gradient separation was used for dead cell removal by centrifugation at 500 × g for 20 min with no brake. Primed T cells were isolated with the negative selection cocktail described above.

### Lipid-Mediated Insertion (Depoting) of Lipid-Conjugated Ligands

Unless specified otherwise (in **Figure 2**), splenocytes, isolated B cells, or isolated T cells were incubated with CpG (5 μM), lipid-CpG (5 μM), Pam2CSK4 (10 μg/mL), or Pam3CSK4 (10 μg/mL) for 1 h in complete RPMI 1640 media supplemented with 10% fetal bovine serum. Splenocytes or T cells were then washed 3 times with PBS plus 1% bovine serum albumin to remove unbound ligand.

Cells were incubated with αCD16/32 antibody (clone 93; Biolegend) to block non-specific antibody binding by Fc receptors for 5 min at 25°C. Cells were stained with the following antibodies: CD4 (clone GK1.5; PerCP/Cyanine5.5), CD8a (clone 53-6.7; APC), and B220 (clone RA3-6B2; PE/Cy7) 15 min at 25°C. PE-conjugated streptavidin was used to bind depoted biotinylated Pam2CSK4. Cell viability was determined by LIVE/DEAD^TM^ exclusion staining per manufacturer's instructions (ThermoFisher).

### TLR2 Ligand Persistence and Bystander Cell Activation Assays

Naïve or primed T cells were rested in complete RPMI 1640 media and supplemented with 10% FBS after depoting with Pam2CSK4 or Pam3CSK4 as described above. At selected time-points (0, 1, 2, 5, or 8 days post-depoting), T cells were washed and fixed with 4% paraformaldehyde (Sigma) for 15 min at 25°C. After washing 5 times with PBS + 1% BSA, at 1,000 × g for 5 min, T cells were cultured in complete RPMI 1640 media and supplemented with 10% FBS. Bystander B cells were added in co-cultures at a 1:1 B cell:T cell ratio. Naïve T cells without fixation were immediately cultured with bystander B cells. After 2 days of incubation at 37°C and 5% CO_2_, B-cell activation was measured by fluorescent staining for MHCII (clone M5/114.15.2; FITC) and CD69 (clone H1.2F3; PerCP/Cy5.5).

### Lipid-Conjugated TLR9 Ligand B-Cell Activation Assay

Isolated B cells from C57/BL6 mice were cultured in complete RPMI 1640 media supplemented with 10% FBS after depoting with lipid-CpG and combined with an equal number (50,000 cells) of isolated CD45.1^+^ B cells. After 2 days of co-culture at 37°C and 5% CO_2_, B cells were identified by B220 (clone RA3-6B2; PE-Cy7) CD45.1 (clone A20; PE) antibody, and B-cell activation was measured by MHCII (clone M5/114.15.2; FITC) and CD69 (clone H1.2F3; PerCP/Cy5.5).

### T-Cell Activation Assays

Naïve T cells were labeled with 5 μM of carboxyfluorescein succinimidyl ester (CFSE, ThermoFisher). Labeled cells were cultured in RPMI 1640 media and supplemented with 10% FBS as well as αCD3/CD28 coated Dynabeads^TM^ (Thermo) at a 1:5 bead to T cell ratio after depoting of lipid-CpG, Pam2CSK4, and/or Pam3CSK4 ligands. Non-depoted T cells were treated with soluble lipid-CpG (5 μM), Pam2CSK4 (10 μg/mL), and/or Pam3CSK4 (10 μg/mL). Cell proliferation was measured by CFSE dilution after 3 days, and cell activation was measured by fluorescent staining for CD25 (clone PC61; PE). Cell proliferation index and division index were calculated using FlowJo LLC software. Proliferation index is defined as the total number of cell divisions divided by the number of divided cells, whereas division index is the total number of cell divisions divided by the number of total original cells (24). Paracrine-enhanced proliferation of bystander T cells was measured as described above, with αCD3/CD28 beads added at a 2:5 bead to T-cell ratio.

### Flow Cytometry and Microscopy Analyses

Fluorescently labeled cells were analyzed on a BD LSRII or Beckman Coulter CyAn ADP flow cytometer. The LSRII flow cytometer consisted of 405, 488, 561, and 640 nm excitation laser lines. The CyAn ADP flow cytometer consists of 405, 488, and 635 nm excitation laser lines. Data were analyzed using FlowJo LLC software v10 (Tree Star Inc.).

Cells loaded with FAM-labeled lipid-CpG were prepared for confocal analysis by blocking with normal goat serum and labeling with early endosome marker-1 (EEA1) (clone C45B10; Cell Signaling Technologies) for 12 h at 4°C. Cells were fluorescently labeled with AF647 anti-rabbit IgG (Invitrogen) and DAPI for 60 min at 25°C. Confocal anaylsis was performed on a TCS SP5 microscope (Leica Microsystems). The excitation laser lines used were 405, 488, and 633 nm. Lipid-CpG and EEA1 pixel intensities were obtained from 8-bit images, and pixels were binned in 2 × 2 matrices after noise subtraction (removing pixels = < 5 and pixels = 255). All imaging analysis was performed using Matlab.

### Enzyme-Linked Immunosorbent Assays (ELISAs) for Lipid-Conjugated Ligand Detection and Cytokine Quantification

One million purified T cells were depoted with FAM-labeled lipid-CpG, biotinylated Pam2CSK4, or biotinylated Pam3CSK4 as described above, and then lysed with Glo lysis buffer (Promega) for 10 min at 25°C. Lysates were collected and plated onto Nunc MaxiSorp ELISA plates (Thermo). After 2 h of incubation at 25°C, FAM-labeled lipid-CpG was excited at 488 nm, and fluorescent emission at 520 nm was quantified by a microplate fluorescence reader. For Pam2CSK4 and Pam3CSK4 samples, horseradish peroxidase (HRP)-labeled streptavidin was added for 2 h incubation at 25°C, and then TMB substrate (Thermo) was added to observe changes in absorbance at 450 nm with a microplate spectrophotometer (BioTek).

T-cell activation was analyzed by collecting supernatants on day 2 post-activation for IL-2, IL-4, and IFNγ quantification by ELISA per manufacturer's instructions (BioLegend).

### Regression and Statistical Analyses

For regression models, a least-squares two-phase decay model was fit to the median fluorescence intensity (MFI) data from the TLR2 ligand persistence assay. Effective half-life, the fast half-life of the two-phase decay, is computed as followed:

$$\begin{array}{l} t_{1/2}=\ln\left(\frac{2}{fast\;rate\;constant}\right) \end{array}$$

Colocalization analysis was performed on confocal micrographs using Pearson's correlation coefficient, ρ, computed as followed:

$$\begin{array}{l} \rho =\frac{\Sigma_{i}\left(x_{i}-\bar{x}\right)\left(y_{i}-\bar{y}\right)}{\sqrt{\Sigma_{i}{\left(x_{i}-x\right)}^{2}\Sigma_{i}{\left(y_{i}-y\right)}^{2}}} \end{array}$$

where *x* and *y* are the pixel values of respective lipid-CpG and EEA1 fluorescence intensities from spatial location denoted by *i*. $\bar{x}$ and $\bar{y}$ are mean pixel intensities of lipid-CpG and EEA1 fluorescence intensities over *i*, respectively. Analysis was performed with Matlab.

One-way, repeated measures (RM) one-way, and two-way analyses of variance (ANOVAs) were performed followed by comparisons using Sidak's correction methods. Comparisons to soluble controls were also performed using one-tailed ratio-paired *t*-tests. All statistical analysis and regression models were performed with Prism software (GraphPad).

## Results

### Lipid-Conjugated TLR Ligands Efficiently Inserted Into Plasma Membranes

**Figure 1:**
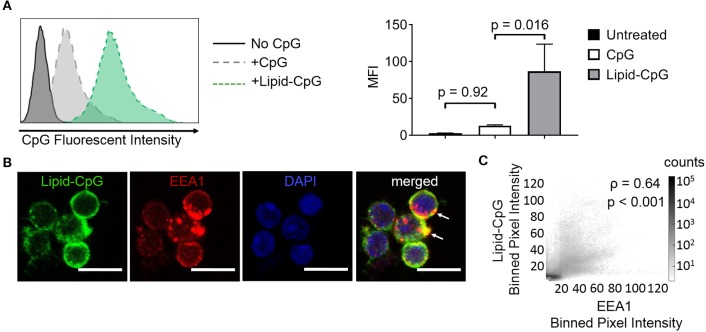
Insertion of TLR9 ligand into murine immune cells is enhanced by lipid tail. Fluorescein (FAM) labeled CpG ODN or diacyl lipid conjugated CpG ODN (lipid-CpG) were incubated with splenic immune cells at 5 μM for 1 h at 37°C in supplemented RPMI media. **(A)** Representative fluorescent intensity as measured by flow cytometry (left), and quantification of fluorescent intensity (right). *p*-values between indicated conditions were determined by one-way ANOVA with Sidak's method for multiple comparisons correction. Data showed m ± s.d. (*n* = 3 independent samples). **(B)** Confocal micrographs showed lipid-CpG delivery to cell plasma membranes and associated with early endosome marker-1 (EEA1) (white arrows). Lipid-CpG (green), EEA1 (red), DAPI (blue). Scale bar = 10 μm. **(C)** Bivariate scatter plot showed binned (2x2) pixel intensities between lipid-CpG and EEA1. Pearson's correlation coefficient was determined as ρ = 0.64 (*p* < 0.001). Data compiled from *n* = 52 cells from 3 independent samples.

The ability of lipids to insert into plasma membranes can be exploited for stable anchoring of lipid-conjugated immunostimulatory TLR ligands into cells. This phenomenon is similar to the natural membrane insertion of GPI-anchored proteins. We have previously conjugated the intracellular TLR9 ligand CpG DNA to a diacyl stearoyl (C18) lipid, termed lipid-CpG, and demonstrated strong association with plasma membranes of cancer cells (10). Here, we tested whether lipid-CpG can also depot into plasma membranes of immune cells. Lipid-CpG incubated for 1 h with resting splenocytes (B cells, CD4^+^ T cells, CD8^+^ T cells) showed increased ligand loading by 6.7-fold compared to free CpG (Figure 1A). Confocal microscopy analysis suggested lipid-CpG associated with early endosomal marker-1 (EEA1) within immune cells (Figure 1B). A bivariate scatter plot showed counts of lipid-CpG and EEA1 pixels from multiple confocal micrographs; colocalization analysis between lipid-CpG and EEA1 pixel intensities demonstrated a Pearson correlation coefficient of ρ = 0.64 (*p* < 0.001), supporting partial colocalization and uptake by endocytosis (Figure 1C) (25). These data demonstrated lipid-mediated cell loading can deliver cargo to cell surface and intracellular compartments, overcoming the challenges of low non-specific uptake generally associated with resting lymphocytes (26). We termed this lipid-mediated insertion “depoting,” reflecting the likely rapid partitioning of lipid moieties into the lipid bilayer of plasma membranes (27, 28).

**Figure 2:**
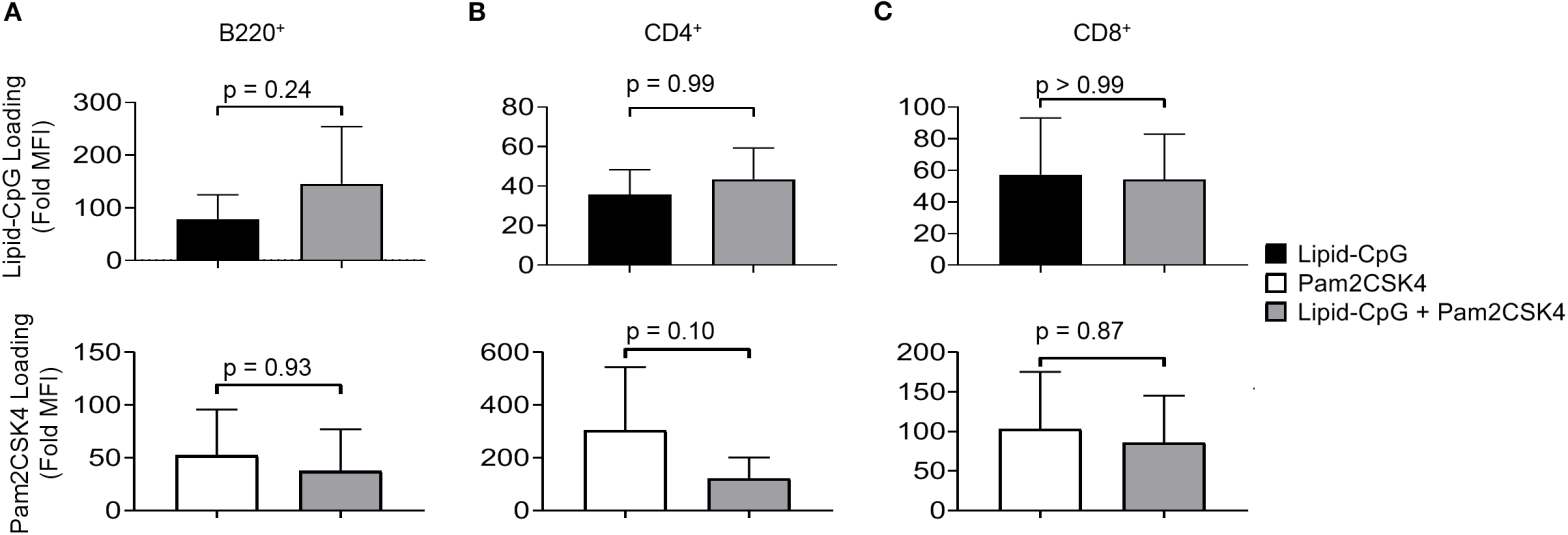
Lipid-tailed ligands efficiently depot alone and together into resting B and T cells. Fluorescently labeled lipid-CpG or biotinylated Pam2CSK4 was incubated either separately or together with splenic immune cells at 5 μM or 10 μg/mL, respectively, for 1 h at 37°C. Splenic immune cells were gated by lineage markers to analyze lipid-CpG (*top row*) and Pam2CSK4 (*bottom row*) depoting in 3 cell types: **(A)** B220^+^ B cells, **(B)** CD4^+^ T cells, and **(C)** CD8^+^ T cells. Loading was calculated as the fold increase of sample median fluorescent intensity (MFI) normalized to non-depoted cells for each ligand. *p*-values between indicated conditions for each panel (top and bottom) were determined by one-way ANOVA with Sidak's method for multiple comparisons correction. Data showed m ± s.d. (*n* = 4 independent samples).

We next investigated whether a cell surface TLR ligand, Pam2CSK4, can also be depoted into splenic immune cells given differences in its diacyl palmitoyl (C16) lipid tail. We also tested depoting of Pam2CSK4 in parallel with lipid-CpG to determine if multiple lipid-tailed ligands can be depoted into cells without membrane saturation. We analyzed lipid-CpG and Pam2CSK4 depoting individually and together in B220^+^ B cells (Figure 2A), CD4^+^ T cells (Figure 2B), and CD8^+^ T cells (Figure 2C). Depoting lipid-CpG and Pam2CSK4 together did not decrease ligand levels compared to ligands depoted alone. These data suggested that delivery of TLR2 and TLR9 ligands can be mediated by C16 or C18 lipid tails, respectively, and that depoting 2 ligands was feasible without loading saturation under tested conditions. Depoting also had no effect on cell viability compared to untreated cells (Figure S1). Overall, our data showed that B and T cells have a high capacity for rapid depoting with multiple lipid-conjugated TLR ligands, and that depoting achieved delivery of ligands into plasma membranes without toxicity to cells.

**Figure 3:**
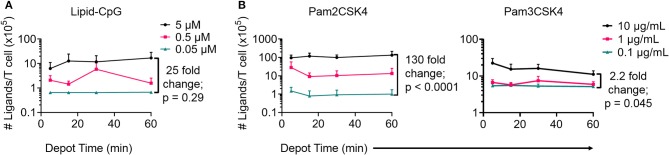
Depoting of TLR ligands provides dose control. Depoted lipid-tailed TLR ligands into polyclonal T cells was dependent on input concentration for **(A)** lipid-CpG; and **(B)** Pam2CSK4 or Pam3CSK4 polypeptides. The fold increase in average ligand per T cell is indicated per ELISA analysis. *p*-values between indicated comparisons as determined by two-way ANOVA with Sidak's tests. Data showed mean ± s.d. (*n* = 3–4 independent samples).

Next, we optimized depoting conditions to more precisely control the abundance of ligands. Enzyme-linked immunosorbent assays (ELISAs) were used to quantify depoted lipid conjugates in purified mouse T cells. Conjugate concentration during depoting was tested over a 100-fold range and depoting times were tested ranging from 5 to 60 min. Cells were lysed after depoting to detect the average number of ligands per T cell. Depoting 5 μM of lipid-CpG for 60 min resulted in 25-fold more molecules per T cell when compared to depoting 0.05 μM of lipid-CpG (Figure 3A).

We then quantified depoting of TLR2 ligands. Increasing depoting concentration of Pam2CSK4 from 0.1 to 10 μg/mL resulted in a 130-fold increase in the number of depoted molecules per T cell (Figure 3B, left). Both lipid-CpG and Pam2CSK4 depoting plateaued after 1 h. Increasing depoting concentration did not change preferential insertion into CD4^+^ or CD8^+^ T cells (Figures S2A,B). Pam3CSK4, a synthetic lipid-peptide with a triacyl palmitoyl lipid, is another well-defined TLR2 ligand (16, 29). We used this ligand to test if the third hydrophobic lipid tail altered depoting in T cells. Pam3CSK4 depoting increased with time, but increasing the depoting concentration of Pam3CSK4 from 0.1 μg/mL to 10 μg/mL resulted in a smaller 2.2-fold increase in number of depoted molecules per T cell (Figure 3B, right) when compared to Pam2CSK4. The diacyl and triacyl TLR2 ligands depoted into T-cell plasma membranes in a dose-dependent manner, suggesting that depoting is largely independent of tail number. We depoted cells in subsequent experiments for 1 h at the highest ligand concentrations above to maximize depoting.

### Depoted Cell-Surface Ligands Induced Paracrine Cell Activation

**Figure 4:**
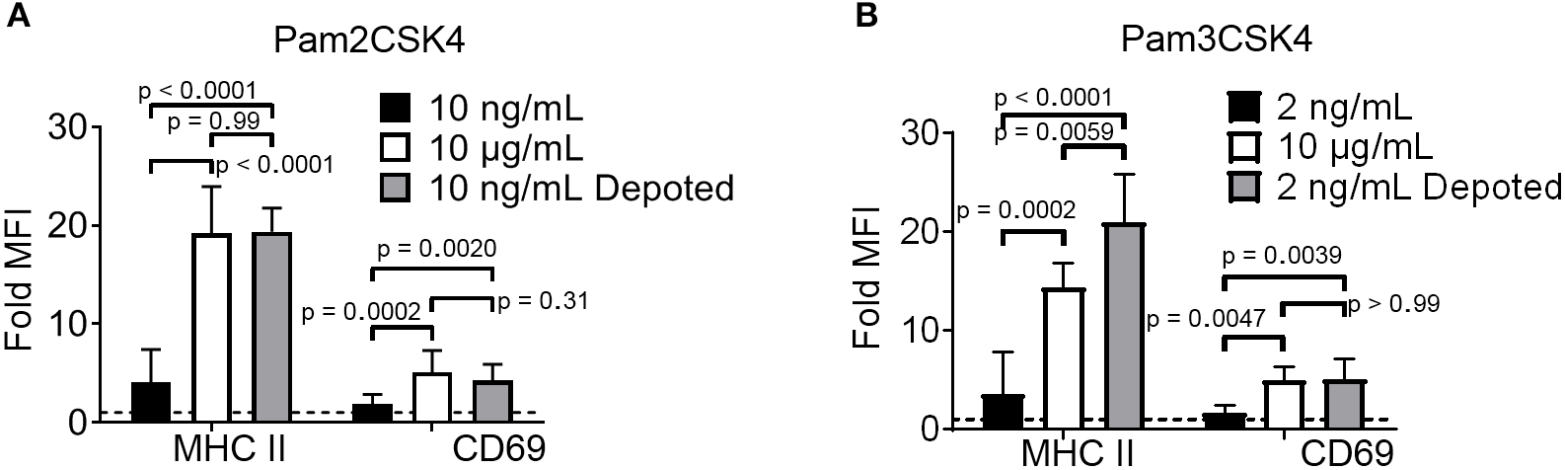
Depoted TLR ligands activate cells through paracrine signaling. Polyclonal T cells were incubated with **(A)** Pam2CSK4 or **(B)** Pam3CSK4 for 1 h under indicated conditions, and then co-cultured with B cells for 2 days. B-cell activation was determined by fold MFI of activation markers, MHC II and CD69, as measured by flow cytometry. Sample MFIs were normalized to unstimulated B cell controls. Dashed line represents theoretical no activation (MFI = 1). *p*-values between indicated conditions were determined by RM one-way ANOVA with Sidak's method for multiple comparisons correction. Data showed m ± s.d. (*n* = 5–6 independent experiments).

TLR2 ligation has successfully enhanced cellular immunity by direct signaling on effector cells (e.g., T cells) and antigen-presenting cells (APCs) (16, 22). We hypothesized that TLR2 depoting can enhance bystander APC functions. To test this, we first validated that TLR2 ligands on T cells can engage its receptor on bystander cells using TLR2^+^ APCs. B cells were chosen as bystander APCs due to their dose-dependent sensitivity to Pam2CSK4 and Pam3CSK4 (Figures S3A,B). T cells depoted with Pam2CSK4 activated B cells after 2 days of co-culture, upregulating MHC II (19-fold) and CD69 (3.8-fold) (Figure 4A) (24). T cells depoted with Pam3CSK4 also activated B cells in co-culture, upregulating MHC II (21-fold) and CD69 (4.4-fold) (Figure 4B). MHC II and CD69 expression levels activated by Pam2CSK4 and Pam3CSK4-depoted T cells were similar to 10 μg/mL of soluble ligand. In fact, depoted Pam3CSK4 induced 1.5-fold higher MHC II expression than 10 μg/mL of soluble Pam3CSK4. This high dose of soluble ligand was 1,000-5,000-fold more concentrated than the estimated dose on depoted T cells (Table S1).

We next tested whether depoting can enhance cell sensitivity to TLR2 signaling per dose of ligand compared to soluble delivery. These doses (10 ng/mL for Pam2CSK4 and 2 ng/mL for Pam3CSK4) were matched to the respective amount of ligand quantified in depoted cells as determined by ELISA (Figures 3B,C, Table S1). T cells depoted with Pam2CSK4 induced higher expression of MHC II (5.3-fold) and CD69 (2.2-fold) when compared to the same dose of soluble Pam2CSK4 (Figure 4A). Depoted Pam3CSK4 induced higher expression of MHC II (6.1-fold) and CD69 (2.8-fold) when compared to the same dose of soluble Pam3CSK4 (Figure 4B). Consistent with our hypothesis, none of the dose-matched soluble Pam2CSK4 and Pam3CSK4 activated B cells. These studies demonstrated that depoted TLR2 ligands were presented to bystander cells, and that cell surface-bound presentation of TLR2 ligands provided stronger signaling than the equivalent ligand dose in solution.

### Intracellular Ligands Activated Depoted Immune Cells but Not Bystanders

**Figure 5:**
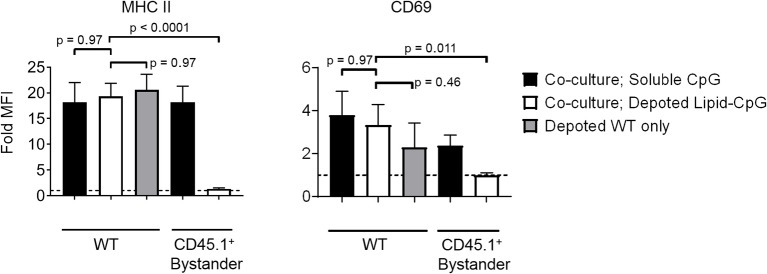
Depoted TLR ligands activate cells through autocrine signaling. Wildtype (WT) B cells were depoted with 5 μM of lipid-CpG and co-cultured with CD45.1^+^ bystander B cells. MHCII and CD69 on both depoted (WT) and bystander (CD45.1^+^) B cells were measured after 2 days of co-culture by flow cytometry and normalized to respective unstimulated B cells. Dashed line represents theoretical no activation (MFI = 1). Indicated *p*-values were determined by RM one-way ANOVA with Sidak's method for multiple comparisons correction. Data showed m ± s.d. (*n* = 4 independent samples).

Bystander cells can be activated by paracrine interactions with depoted cells, as well as from release of depoted ligands over time into solution. We used lipid-CpG, a TLR9 ligand that must be internalized for signaling, to test whether release of depoted ligand over time contributed to paracrine cell activation. A co-culture assay was used with B cells as both depoted cells and bystander cells given their sensitivity to CpG stimulation (30). Lipid-CpG was depoted into B cells from wild-type mice, and then cocultured with congenic CD45.1^+^ bystander B cells for 2 days. Lipid-CpG depoted B cells showed increased expression of MHC II (19-fold) and CD69 (3.3-fold) when normalized to unstimulated B cells (Figure 5). Levels of MHC II and CD69 were similar to those induced by a high dose of free CpG (5 μM) in solution. This confirmed that depoted ligand can be internalized to provide autocrine stimulation. We then analyzed activation of bystander cells to determine whether paracrine signaling occurred through soluble ligand release. Bystander CD45.1^+^ B cells were not activated, with activation marker levels comparable to unstimulated controls (Figure 5). We analyzed whether ligand can spontaneously transfer from plasma membranes of depoted cells to undepoted bystanders. The presence of bystander B cells did not decrease activation of depoted B cells since MHC II and CD69 expression levels in co-cultures were comparable to expression on depoted cells cultured alone. These data demonstrated that depoted ligand remained stably compartmentalized in depoted cells, and that lipid-CpG was not released at functional levels into solution or transferred to plasma membranes of bystander cells.

### Surface Presentation of Depoted TLR2 Ligands Was Non-permanent

**Figure 6:**
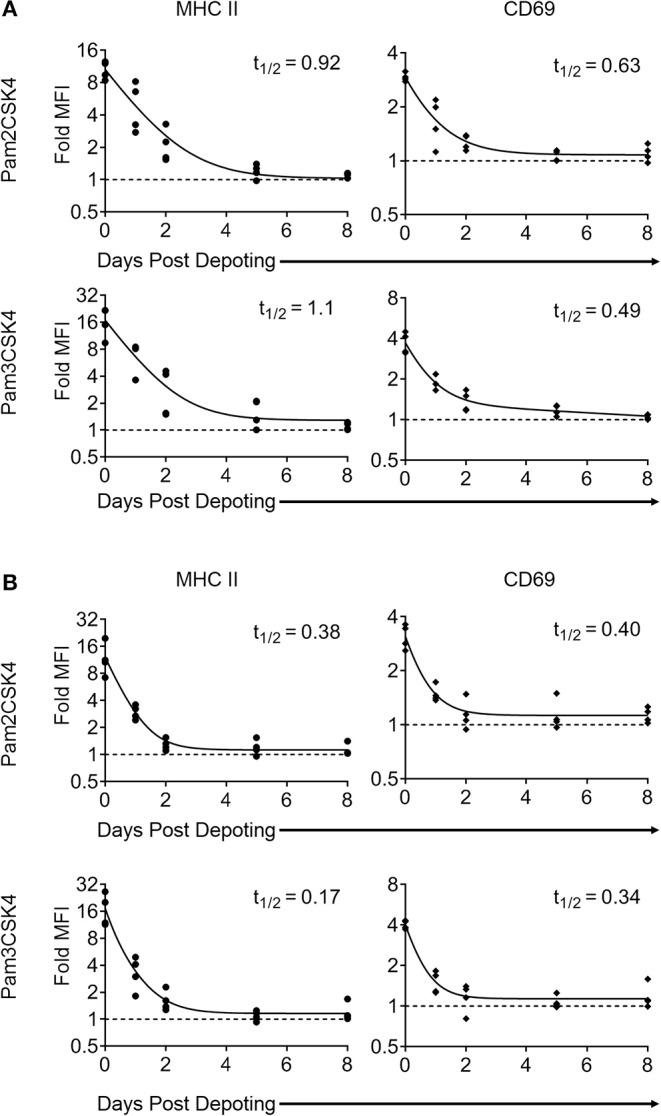
Surface presentation of depoted TLR2 ligands on T cells is non-permanent. Splenocytes were cultured either **(A)** (as naïve T cells) in absence of, or **(B)** (as primed T cells) in presence of 2 μg/mL of Concanavalin A and 10 ng/mL of IL-7 for 2 days. Purified T cells were then depoted with either Pam2CSK4 or Pam3CSK4 for 1 h at 37°C in supplemented RPMI media. T cells were then rested in IL-7 for 0, 1, 2, 5, or 8 days. At each time-point, cells were fixed and co-cultured for 2 days with purified B cells. B-cell activation was determined by fold MFI of MHC II (circle) and CD69 (diamond) with respect to unstimulated controls. Dashed line represents theoretical no activation (MFI = 1). Solid lines are two-phase decay non-linear regression curves as determined by independent samples shown. Effective fast half-life in days (t_1/2_) as shown. *n* = 3–4 independent samples.

Depoting is a non-permanent cell modification, so we next characterized the persistence of depoted ligands on immune cell surfaces. Purified T cells were depoted with TLR2 ligands, and then rested for up to 8 days in IL-7. After resting, cells were fixed in 4% paraformaldehyde to preserve persisting surface TLR2 ligands. The persistence of surface TLR2 ligands was determined by co-culturing fixed T cells with bystander B cells. Low level IL-7 supplementation was used to sustain T-cell viability without eliciting cell proliferation over the 8-day rest period (Figure S4). We analyzed MHC II and CD69 levels on B cells induced by T cells fixed immediately after depoting (0-day) and determined levels were comparable to levels observed in Figure 3. This verified that fixation did not alter the recognition of TLR2 ligand. Levels of surface-bound ligand decayed over time, with the shortest effective fast half-life of 0.49 days for Pam3CSK4 induction of CD69 (Figure 6A). Overall, bystander cell activation showed that surface-presented ligand functionally persisted between 2 and 4 days post-depoting.

Depoted ligands are not permanently persistent on cell surfaces, so their efficacy could be diluted as T cells divide. We used proliferating T cells to determine whether cell division causes faster decay of depoted ligands. Naïve T cells were primed for 2 days with concanavalin A and IL-7 before depoting. Primed cells were depoted with Pam2CSK4 or Pam3CSK4 and rested for up to 8 days. Primed T cells retained viability for 8 days in IL-7, similar to naïve T cells (Figure S4A, right). Depoted Pam2CSK4 and Pam3CSK4 decayed at a similar rate on primed T cells compared to naïve T cells, activating B cell bystanders as determined by MHC II and CD69 expression (Figure 6B). The shortest effective fast half-life of 0.17 days was observed for MHC II levels stimulated by Pam3CSK4. Altogether, our data demonstrated that depoted TLR2 ligands persisted on plasma membranes of both naïve and primed T cells, providing functional paracrine signaling to bystander cells for multiple days. Proliferation of primed T cells did not further increase the surface decay rate for depoted ligands.

### Depoted Lipid-Conjugated TLR2 Enhanced T Cell Activation

**Figure 7:**
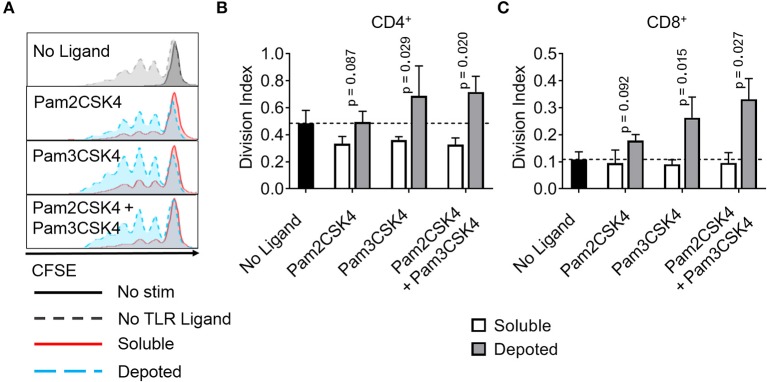
Depoted TLR2 ligands enhance proliferation of activated murine T cells. Purified polyclonal T cells were stained with 5 μM of carboxyfluorescein succinimidyl ester (CFSE). Different combinations of cell surface ligands (Pam2CSK4 and Pam3CSK4) were either directly added in solution (soluble) or depoted into polyclonal T cells for 1 h at 37°C and cultured with αCD3/CD28 beads for 3 days. **(A)** Representative histograms of CD4^+^ T-cell proliferation from delivery of lipid-TLR2 ligand as measured by CFSE dilution. Quantification of division index of **(B)** CD4^+^ and **(C)** CD8^+^ T cells in bulk polyclonal T cells. Dashed lines represent respective averages (mean) of “No Ligand” conditions. *p*-values by between soluble vs. depoted ligands as determined by one-tailed ratio paired *t*-test. Data showed m ± s.d. (*n* = 3 independent samples).

T cells do not constitutively express abundant levels of TLR2 or TLR9, but activated T cells rapidly increase TLR expression (31). Previous studies demonstrate TLR ligands can enhance T cell activation (16, 17, 32). Here, we tested whether depoting TLR ligands in T cells can enhance activation by increased proliferation. We added TLR ligands in solution or depoted into pan CD4^+^ and CD8^+^ T cells from wild-type C57BL/6J mice. T cells were stimulated with αCD3/CD28-coated beads for 3 days, then analyzed for proliferation by flow cytometry. Depoting of TLR2 ligands induced more CD4^+^ T-cell proliferation compared to dose-matched soluble ligands (Figure 7A). Depoting of Pam3CSK4 alone increased CD4^+^ and CD8^+^ T-cell division indices by 1.9- and 2.9-fold, respectively, compared to soluble Pam3CSK4 (Figures 7B,C). Depoting both TLR2 ligands increased CD4^+^ and CD8^+^ T-cell division indices by 2.2-fold and 3.3-fold, respectively, compared to soluble ligands. No lipid-conjugated ligands enhanced CD4^+^ T-cell proliferation index by depoting, but depoted Pam3CSK4 did enhance CD8^+^ T-cell proliferation index (Figure S5). Altogether, these data demonstrated that Pam3CSK4, alone and in combination with other lipid-conjugated ligands, provided co-stimulation during T-cell division.

### Depoted TLR2 Ligands Enhanced T-Cell Activation by Paracrine Signaling

We tested whether depoting with TLR9 ligand can enhance T-cell activation and functions. Lipid-CpG did not increase proliferation of CD4^+^ or CD8^+^ T cells above levels compared to those without ligand. Combining lipid-CpG with Pam2CSK4 or Pam3CSK4 did not enhance CD4^+^ T-cell proliferation. CD8^+^ T-cell proliferation did increase with depoted lipid-CpG and Pam3CSK4 (Figure S6), but these levels were lower than T cells depoted with Pam3CSK4 alone, as shown in Figure 7C or Figure S5B. We determined how depoting with TLR2 and TLR9 ligands affected cytokine secretion by analyzing IL-2 and IL-4 levels as signature Th1 and Th2 cytokines, respectively. Soluble TLR9 ligand elicited detectable IL-4 production, while depoted TLR9 ligand decreased IL-4 levels below the limit of detection (Figure S7A). Soluble and depoted TLR2 ligands resulted in IL-4 levels below the limit of detection. Depoting of TLR2 and TLR9 ligands resulted in higher IL-2 levels compared to dose-matched soluble ligands (Figure S7B). These data suggested that co-stimulation of T cells by depoted TLR ligands elicited a more pro-inflammatory Th1-like phenotype.

**Figure 8:**
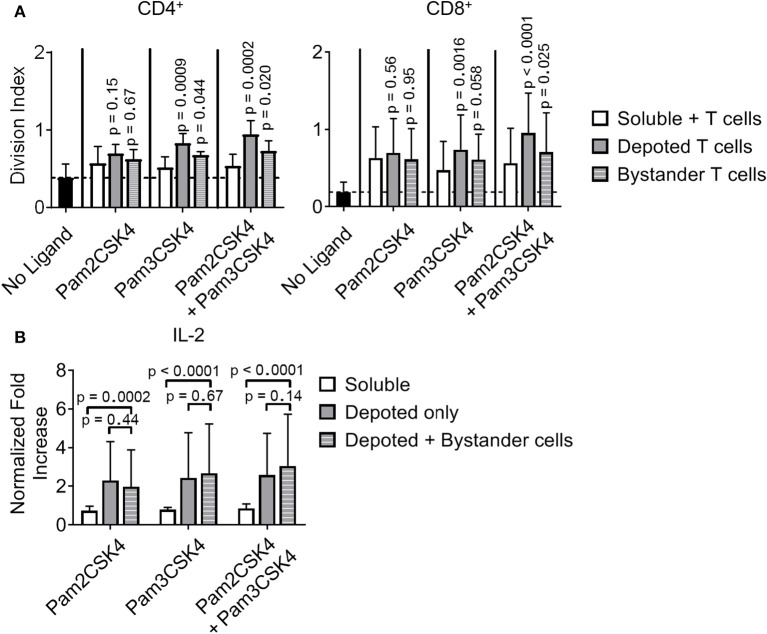
Depoted TLR2 ligands enhance murine T-cell activation by increased cell division and inflammatory IL-2 secretion. **(A)** Purified polyclonal T cells were stained with 5 μM of carboxyfluorescein succinimidyl ester (CFSE). Different combinations of cell surface ligands (Pam2CSK4 and Pam3CSK4) were either directly added in solution (soluble) or depoted into polyclonal T cells, and co-cultured with non-depoted T cells and αCD3/CD28 beads for 3 days. Quantification of division index of CD4^+^ and CD8^+^ T cells in bulk polyclonal T cells. Dashed lines represent respective averages (mean) of “No Ligand” conditions. *p*-values within each group were determined by comparing with respective soluble ligand as determined by RM one-way ANOVA with Sidak's method for multiple comparisons correction. Data showed m ± s.d. (*n* = 5 independent samples). **(B)** IL-2 from cell supernatants was measured by ELISA on day 2. Concentrations were normalized to αCD3/CD28 bead-stimulated T cells in the absence of TLR2 ligand. *p*-values between indicated pairwise comparisons were determined by two-way ANOVA with Sidak's method for multiple comparisons correction. Data showed m ± s.d. (*n* = 5 independent samples).

While depoting increased T-cell proliferation, we next analyzed whether depoted TLR2 ligands provide autocrine co-stimulation or paracrine co-stimulation to bystander T cells. Depoted T cells were mixed with non-depoted bystander T cells, and co-cultures were stimulated with αCD3/CD28 beads for 3 days. CD4^+^ and CD8^+^ T-cell division indices of Pam3CSK4-depoted cells increased 1.6-fold compared to respective soluble ligand (Figure 8A). *Cis*-depoted Pam2CSK4 and Pam3CSK4 (depoted onto the same cell) increased division indices of depoted cells: 1.8-fold for CD4^+^ T cells and 1.7-fold for CD8^+^ T cells compared to soluble ligands. No changes were observed in proliferation indices (Figure S8). Pam3CSK4-depoted T cells also increased bystander CD4^+^ and CD8^+^ T-cell division indices by 1.3-fold compared to soluble ligand. *Cis-*depoted Pam2CSK4 and Pam3CSK4 increased division indices of bystander cells by 1.4-fold for CD4^+^ T cells and 1.3-fold for CD8^+^ T cells compared to soluble ligands. These data demonstrated that TLR2 ligand depoted T cells can engage in autocrine and paracrine enhancement of proliferation, activating themselves as well as bystander T cells. We analyzed whether depoted T cells can enhance cytokine secretion by paracrine engagement of bystander T cells. No differences in secretion of Th1 cytokines, IFNγ or IL-2, were observed with addition of bystander T cells to depoted T cells (Figure 8B, Figure S9A). These data showed that depoted TLR2 ligands enhanced bystander T-cell proliferation but not Th1 cytokine secretion.

However, depoting of TLR2 ligands increased IFNγ and IL-2 secretion levels when compared to dose-matched soluble ligands. Depoting TLR2 ligands did not increase IFNγ levels (Figure S9A), but did increase IL-2 levels and expression levels of the IL-2 receptor, CD25 (Figure 8B, Figure S9B). Depoting with Pam2CSK4 induced a 2.7-fold increase IL-2 levels compared to soluble ligand (Figure 8B). Depoting with Pam3CSK4 induced a 3.4-fold increase in IL-2 levels compared to soluble ligand. *Cis*-depoted Pam2CSK4 and Pam3CSK4 induced a 3.4-fold increase in IL-2 levels compared to soluble ligands. These data again demonstrated that surface-bound presentation of depoted TLR2 ligands provided stronger signaling than dose-matched soluble ligands through enhanced IL-2 secretion.

## Discussion

In this study, we characterized depoting of lipid conjugates as a novel method for simple, non-genetic engineering of immune cells. Our data showed diacyl C16 and C18, and triacyl C16 lipid tails conjugated to DNA and protein cargoes, and depoted into plasma membranes in a concentration-dependent manner. We demonstrated the potential to engineer cells with TLR2 and TLR9 ligands targeting surface and intracellular receptors, respectively. Depoted TLR2 ligands enhanced bystander B- and T-cell activation when compared to dose-matched soluble ligands. Depoted TLR9 ligands showed autocrine activation of depoted B cells with minimal paracrine activation of bystanders, demonstrating delivery of depoted ligands with minimal release into solution or transfer between plasma membranes. Depoting stored detectable levels of surface-bound ligands on resting and proliferating T cells for bystander cell activation for similar durations, up to 4 days of *in vitro* co-culture. We also demonstrated multiplex loading of 2 distinct ligands onto the same plasma membranes without reaching loading saturation or decreasing cell viability. Altogether, our results demonstrated lipid-mediated depoting is a facile, modular drug delivery platform for diverse ligands that target receptors in distinct subcellular spaces.

Depoting TLR ligands can provide precise control over ligand dose by changing input ligand concentration. Varying the depoting concentration of lipid-CpG and Pam2CSK4 over a 100-fold range resulted in 25- and 130-fold increases, respectively, of detected ligand on cells. This suggests that changing depoting concentrations can precisely control drug dosing, a feature that can dictate whether a drug induces immunogenic or tolerogenic responses (33, 34). We determined that cells can be loaded by multiplexing with 2 depoted ligands and have no detectable loading saturation. However, differential loading levels on B and T cells in Figure 2 suggest that lymphocyte subsets have distinct plasma membrane compositions, including TLR9 ligand-internalization receptors that may skew immune cell surface binding and alter total depoting capacity in a ligand- and cell-dependent manner (35, 36). Here, we demonstrated depoting of >1 million molecules per cell for some ligands, which is classified as very abundant based on recent estimates (37). This suggests that depoting loads a high absolute amount of cargoes onto plasma membranes beyond the likely capacity of just surface receptor binding.

We also demonstrated that the lipids we used (i.e., diacyl/triacyl C16 and diacyl C18 tails) effectively delivered ligands to plasma membranes. Our previous study showed that a single acyl lipid tail is inefficient at TLR ligand delivery (7, 10). Here, our results demonstrated that two and three-tailed lipids depoted efficiently. Thus, multiple lipid tails are a key design parameter for membrane delivery, and may be a simple way to enhance delivery of cargo to plasma membranes. One explanation of lipid-mediated insertion of TLR2 lipid-ligands, i.e., Pam2CSK4 and Pam3CSK4, into plasma membranes is the spontaneous formation of micelles when delivered at a high ligand concentration. This may result in particulate-mediated fusion with plasma membranes. However, the critical micelle concentrations of Pam2CSK4 and Pam3CSK4 are much higher than our input concentrations of 10 μg/mL (38). Our hypothesis of lipid-ligand “depoting” is a likely explanation that is supported by lipid-mediated partitioning of plasma membranes (27, 28) and the natural membrane insertion of GPI-anchored proteins. Altogether, depoting is advantageous not only for delivering synthetic TLR2 and TLR9 ligands as shown in our study, but may also apply to other potent immunostimulatory TLR ligands with greater than three lipid tails, including TLR4 ligands.

The controlled delivery of autocrine or paracrine signaling is critical to the further understanding of immunological signaling and enhancing cell-based therapies. Depoted Pam3CSK4 induced higher levels of MHC II on APCs than a soluble dose that was 5,000-fold larger, suggesting the depoted cells may increase antigen presentation by bystander APCs. This increase in cell activation highlights the enhanced potency of surface presented TLR2 ligands compared to the same dose of ligand delivered in solution. We also demonstrated that depoting of a cell surface ligand enhanced other lymphocyte functions; depoting TLR2 ligands increased CD4^+^ and CD8^+^ T-cell division, and promoted inflammatory responses of bystander T cells. This simple method of delivery can be leveraged in the clinic to activate TLR2 in cytotoxic cells, including T cells and NK cells (22, 39).

In cancer, prolonging the persistence of infused T cells while also eliciting inflammatory responses by promoting endogenous anti-tumor immune cell functions can enhance therapeutic efficacy. However, paracrine signaling of delivered adjuvants is not always desirable for promoting anti-tumor responses (40, 41). Our delivery platform also demonstrated exclusive autocrine signaling with an intracellular ligand by targeting cell endosomes while avoiding paracrine and bystander activation. Analysis of the correlation between lipid-CpG and EEA1 fluorescent intensities showed a positive Pearson's correlation coefficient (ρ = 0.64), suggesting partial colocalization between lipid-ligand and endosomes after uptake (25). Since the correlation of lipid-ligand is not perfect with early endosomes, lipid-mediated depoting likely traffics to multiple intracellular compartments, which may provide unique opportunities for drug delivery. Extensive studies have demonstrated engineered systems to deliver drugs, including vaccines, genomic DNA, and anti-cancer cargoes to intracellular compartments (42–46). Lipid-mediated delivery has the potential to deliver cargoes to multiple compartments due to the natural partitioning of membrane lipids. For example, lipid-conjugated vaccine antigen and adjuvant demonstrated increased therapeutic efficacy in treating tumor models (7, 47). This may partially be due to the increased efficiency of antigen delivery to MHC class I and II pathways to prime CD4^+^ and CD8^+^ T cells. The ability to deliver antigen to these pathways for any cell type may reveal new opportunities for antigen-specific immunotherapy development, such as use of non-traditional APCs.

The use of genetically modified cells for cell-based immunotherapies has been one of the most promising, fastest-developing therapeutic approaches in recent years. However, there is a critical need to improve efficacy while reducing toxicity (48, 49). In 2017, the FDA approved 2 adoptive T-cell immunotherapies using chimeric antigen receptors (CARs) for treating cancer, with hundreds of other T-cell therapies currently in clinical trials in cancer and other chronic diseases (50). The addition of more co-stimulatory signals, other adjuvants, or signaling domains to CARs by genetic additions has become an increasing trend in the field (51–53). However, the uncontrolled release of genetically encoded inflammatory cytokines or other adjuvants from perpetually activated CAR T cells presents a new challenge for toxicity management: lack of dose control (49, 54). New CAR technologies will likely entail even more genetic additions, which invariably will carry genetically-related adverse responses and toxicities. These genetic encodings prolong this adverse risks over the lifetime of the cell (55, 56). Expanding genetic modifications to future therapeutic genes is also limited by the packaging capacity of viral vectors (57). We propose that depoting can address these challenges by complementing genetic engineering and providing non-permanent delivery of co-stimulatory signals for 2–4 days. While depoted cargoes are inherently transient, this time window can be critical for therapeutic efficacy. For example, CAR T cells can initially kill a large proportion of target cells within 2-3 days post-infusion (58, 59). Further, Pam3CSK4 can reactivate latently infected HIV reservoirs in patients to enhance recognition and eradication of infected cells (60). TLR2 surface presentation by depoted T cells may enhance this “shock and kill” response by localizing ligand activity and enhancing ligand potency of cytotoxic T cells to reactivate the latent reservoir. Rational selection and combination of depoted vs. genetically engineered features can improve CAR T-cell persistence and efficacy *in vivo* while minimizing toxicities that may constrain clinical efficacy.

Drug delivery strategies for cellular engineering, from gene editing to nanoparticle and lipid carriers, are under continuous and rapid development (1, 2, 61). For example, nanoparticles have been used in multiple formulations to boost cellular therapies by delivering drugs with immune cells *in vivo* (62–64). Previous studies have shown palmitoyl lipid-tailed conjugates of CD28 ligand or IL-2 cytokine can be “painted” onto T cell surfaces without nanoparticles to enhance cell function (8, 9). Our study significantly expands upon these results by demonstrating the modularity of lipid-mediated delivery for different tail lengths and numbers, and showing that delivery of lipid-ligands can be achieved to cell surface and intracellular receptors. A current limitation of lipid-mediated depoting is the transient nature of the ligand on the cell surface, which will limit duration of ligand signaling on therapeutic cells against chronic diseases such as cancer. Future studies should focus on testing this cell engineering system in applications *in vivo*, as well as on determining design criteria to effectively depot other ligand combinations with longer persistence or higher abundance to optimize immunogenic or immunosuppressive effects in disease models.

Our data prove that depoting can engineer diverse immune cell types and expand existing knowledge on lipid dynamics that can be used for designing future drug delivery approaches. We used two innate TLRs targeted to directly enhance T-cell immunotherapies, and showed enhanced T-cell responses with depoted lipid-TLR ligands. This enables re-examination of pathways for T-cell activation that have been previously understudied due to delivery barriers. Rational selection of more clinically potent ligands for autocrine or paracrine signaling will illustrate the utility of depoting in a broad array of cell-based therapies.

## Data Availability Statement

The datasets generated for this study are available on request to the corresponding author.

## Ethics Statement

The animal study was reviewed and approved by UMBC Institutional Animal Care and Use Committee (OLAW Animal Welfare Assurance D16-00462).

## Author Contributions

MZ and GLS conceptualized the study, analyzed the data, and wrote the manuscript. MZ, ES, GS, and GLS designed the experiments. MZ, ES, GS, and DW performed the experiments. CY and HL contributed experimental resources and methods. All authors participated in reviewing and editing the manuscript.

## Conflict of Interest

GLS, MZ, and ES are inventors on a pending US patent application 62/542,842 submitted by University of Maryland, Baltimore County, describing cell engineering by lipid-mediated depoting, which are described in this manuscript. The remaining authors declare that the research was conducted in the absence of any commercial or financial relationships that could be construed as a potential conflict of interest.

## Supplementary Material

Figure S1 Viability of murine immune cells is not decreased after TLR ligand loading. Cell viability of each treatment group was measured by LIVE/DEAD^TM^ exclusion staining. *p*-values when compared to “Untreated” condition were determined by one-way ANOVA and Sidak's method for multiple comparisons correction. Data showed m ± s.d. (*n* = 3 independent samples).

Figure S2 Depoting concentration does not preferentially affect loading into T cells. Increasing concentration of lipid-tailed TLR ligands does not change CD4^+^ or CD8^+^ T-cell depoting ratio for **(A)** lipid-CpG, and **(B)** Pam2CSK4. Median fluorescent intensity (MFI) ratio was determined by dividing the MFI of fluorescent ligand on CD4^+^ T cells by that of CD8^+^ T cells. Data represented mean ± s.d. (*n* = 3 independent samples).

Figure S3 B cells activate in presence of soluble TLR2 ligand in a dose-dependent manner. **(A)** Dose response of Pam2CSK4 and Pam3CSK4 in B-cell culture for 2 days was measured by fold MFI of B cells. Data represented mean ± s.d. (*n* = 3 independent samples). **(B)** Dose response of Pam2CSK4 and Pam3CSK4 in B- and T-cell culture for 2 days was measured by fold MFI of B cells. Data represented mean ± s.d. (*n* = 2 independent samples).

Figure S4 Viability of purified T cells is unchanged after addition of IL-7. Cell viability of T cells that were **(A)** in absence of (naïve T cells, left), or in presence of (primed T cells, right) 2 μg/mL of Concanavalin A and 10 ng/mL of IL-7 for 2 days and then resting in 10 ng/mL of IL-7 for up to 8 days. **(B)** Cell viability of T cells that were without IL-7. Data showed m ± s.d (*n* = 1–2 independent samples).

Figure S5 Depoted TLR2 ligands do not enhance proliferation indices of activated CD4^+^ T cells, but do of CD8^+^ T cells. Purified polyclonal T cells were stained with 5 μM of carboxyfluorescein succinimidyl ester (CFSE). Different combinations of cell surface ligands (Pam2CSK4 and Pam3CSK4) were either directly added in solution (soluble) or depoted into polyclonal T cells for 1 h and cultured with αCD3/CD28 beads for 3 days. Quantification of proliferation indices of **(A)** CD4^+^ and **(B)** CD8^+^ T cells in bulk polyclonal T cells as measured by CFSE dilution. Dashed lines represent respective averages (mean) of “No Ligand” controls. *p*-values by between soluble vs. depoted ligands as determined by one-tailed ratio paired *t*-test. Data showed m ± s.d. (*n* = 3 independent samples).

Figure S6 Depoted lipid-TLR9 ligand does not enhance proliferation of activated T cells. Purified polyclonal T cells were stained with 5 μM of carboxyfluorescein succinimidyl ester (CFSE). Different combinations of TLR2 ligands (Pam2CSK4 and Pam3CSK4) and TLR9 ligand (lipid-CpG) were either directly added in bulk solution (soluble) or depoted into polyclonal T cells for 1 h and cultured with αCD3/CD28 beads for 3 days. **(A)** Representative histograms of CD4^+^ T-cell proliferation from delivery of lipid-TLR ligand as measured by CFSE dilution. Quantification of division and proliferation indices of **(B)** CD4^+^ and **(C)** CD8^+^ T cells in bulk polyclonal T cells as measured by CFSE dilution. Dashed lines represent respective averages (mean) of “No Ligand” controls. *p*-values by between soluble vs. depoted ligands were determined by one-tailed ratio paired *t*-test. Data showed m ± s.d. (*n* = 3 independent samples).

Figure S7 Depoted TLR ligands promote Th1-based T-cell response. Different combinations of TLR2 ligands (Pam2CSK4 and Pam3CSK4) and TLR9 ligand (lipid-CpG) were either directly added in solution (soluble) or depoted into polyclonal T cells for 1 h and cultured with αCD3/CD28 beads for 3 days. Quantification of **(A)** IL-4 and **(B)** IL-2 levels in T-cell supernatents as measured by ELISA. Dashed lines represent limit of detection for respective cytokine detection. (*n* = 1–3 independent samples).

Figure S8 Depoted TLR2 ligands do not enhance cell proliferation indices. Purified polyclonal T cells were stained with 5 μM of carboxyfluorescein succinimidyl ester (CFSE). Different combinations of TLR2 ligands (Pam2CSK4 and Pam3CSK4) were either directly added in bulk solution (soluble) or depoted into stained T cells for 1 h and cultured with non-depoted, stained T cells and αCD3/CD28 beads for 3 days. Quantification of proliferation index of CD4^+^ and CD8^+^ T cells in bulk polyclonal T cells as measured by CFSE dilution. Dashed lines represent respective averages (mean) of “No Ligand” controls. *p*-values within each group is determined by comparing with respective soluble ligand were determined by one-way ANOVA with Sidak's method for multiple comparisons correction. Data showed m ± s.d. (*n* = 5 independent samples).

Figure S9 Depoted TLR2 ligands increase CD25 expression on activated T cells. Purified polyclonal T cells were stained with 5 μM of carboxyfluorescein succinimidyl ester (CFSE). Different combinations of TLR2 ligands (Pam2CSK4 and Pam3CSK4) and TLR9 ligand (lipid-CpG) were either directly added in bulk solution (soluble) or depoted into polyclonal T cells and cultured with αCD3/CD28 beads for 3 days. **(A)** IFNγ from cell supernatants were measured by ELISA on day 2. Concentrations were normalized to αCD3/CD28 bead-stimulated T cells in the absence of TLR2 ligand. *p*-values between indicated comparisons were determined by two-way ANOVA with Sidak's tests; Data depict m ± s.d. (*n* = 4 independent samples). **(B)** CD25 expression as measured by MFI. *p*-values between corresponding soluble vs. depoted ligands as determined by two-way ANOVA with Sidak's multiple comparisons test; Data showed m ± s.d. (*n* = 3 independent samples).

Table S1 Quantitation of cell surface TLR2 ligands.
