# Supplementary figures and images for: Lipid-Mediated Insertion of Toll-Like Receptor (TLR) Ligands for Facile Immune Cell Engineering
Source: Front Immunol. 2020 Apr 22;11:560. doi: 10.3389/fimmu.2020.00560 (PMC7212467; doi:10.3389/fimmu.2020.00560)

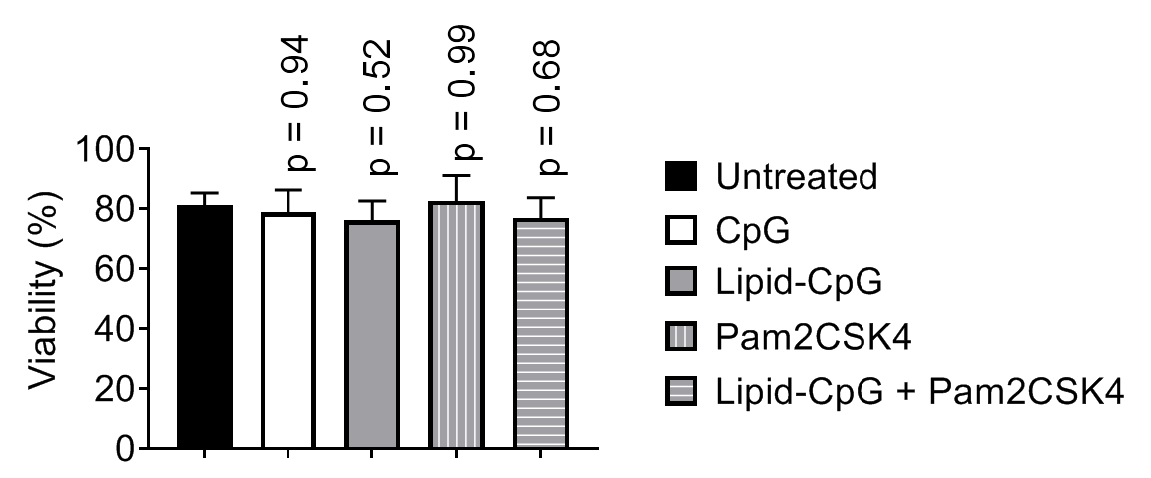

Supplement: Figure S1 — Viability of murine immune cells is not decreased after TLR ligand loading. Cell viability of each treatment group was measured by LIVE/DEADTM exclusion staining. p-values when compared to “Untreated” condition were determined by one-way ANOVA and Sidak's method for multiple comparisons correction. Data showed m ± s.d. (n = 3 independent samples). [file Image_1.TIF]

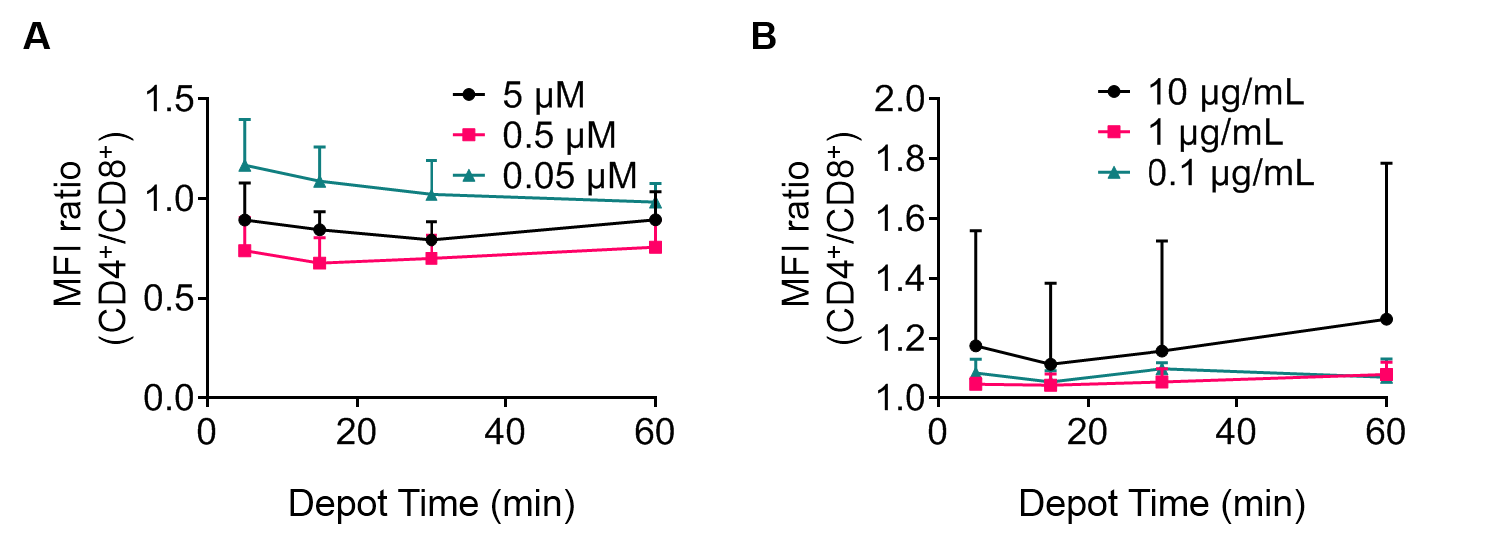

Supplement: Figure S2 — Depoting concentration does not preferentially affect loading into T cells. Increasing concentration of lipid-tailed TLR ligands does not change CD4+ or CD8+ T-cell depoting ratio for (A) lipid-CpG, and (B) Pam2CSK4. Median fluorescent intensity (MFI) ratio was determined by dividing the MFI of fluorescent ligand on CD4+ T cells by that of CD8+ T cells. Data represented mean ± s.d. (n = 3 independent samples). [file Image_2.TIF]

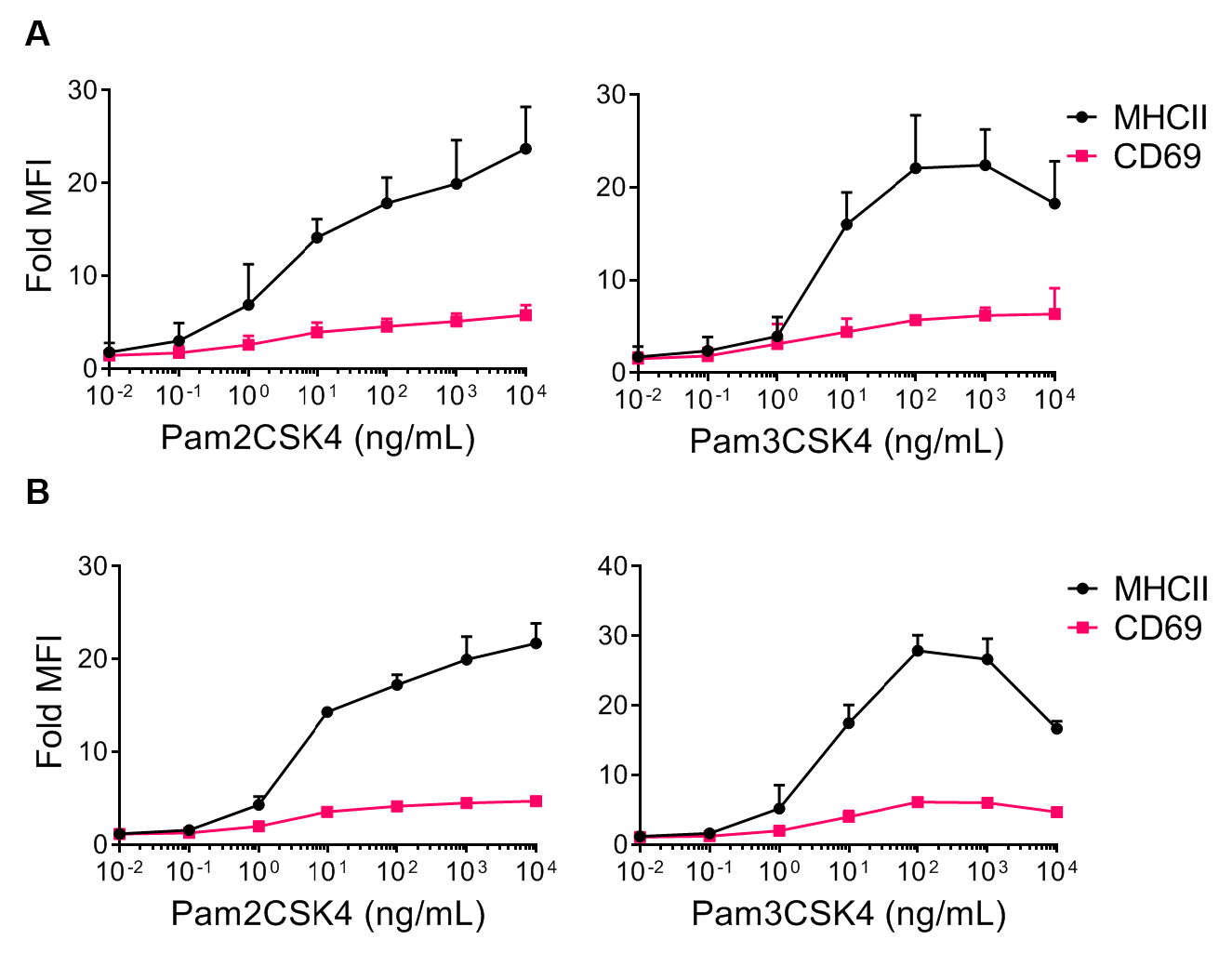

Supplement: Figure S3 — B cells activate in presence of soluble TLR2 ligand in a dose-dependent manner. (A) Dose response of Pam2CSK4 and Pam3CSK4 in B-cell culture for 2 days was measured by fold MFI of B cells. Data represented mean ± s.d. (n = 3 independent samples). (B) Dose response of Pam2CSK4 and Pam3CSK4 in B- and T-cell culture for 2 days was measured by fold MFI of B cells. Data represented mean ± s.d. (n = 2 independent samples). [file Image_3.TIF]

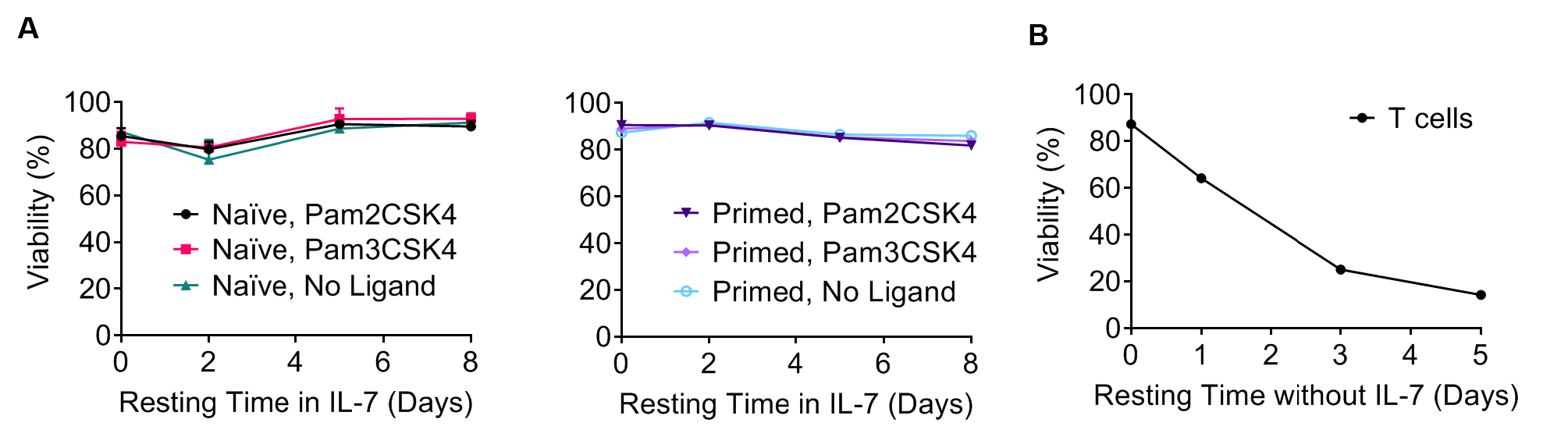

Supplement: Figure S4 — Viability of purified T cells is unchanged after addition of IL-7. Cell viability of T cells that were (A) in absence of (naïve T cells, left), or in presence of (primed T cells, right) 2 μg/mL of Concanavalin A and 10 ng/mL of IL-7 for 2 days and then resting in 10 ng/mL of IL-7 for up to 8 days. (B) Cell viability of T cells that were without IL-7. Data showed m ± s.d (n = 1–2 independent samples). [file Image_4.TIF]

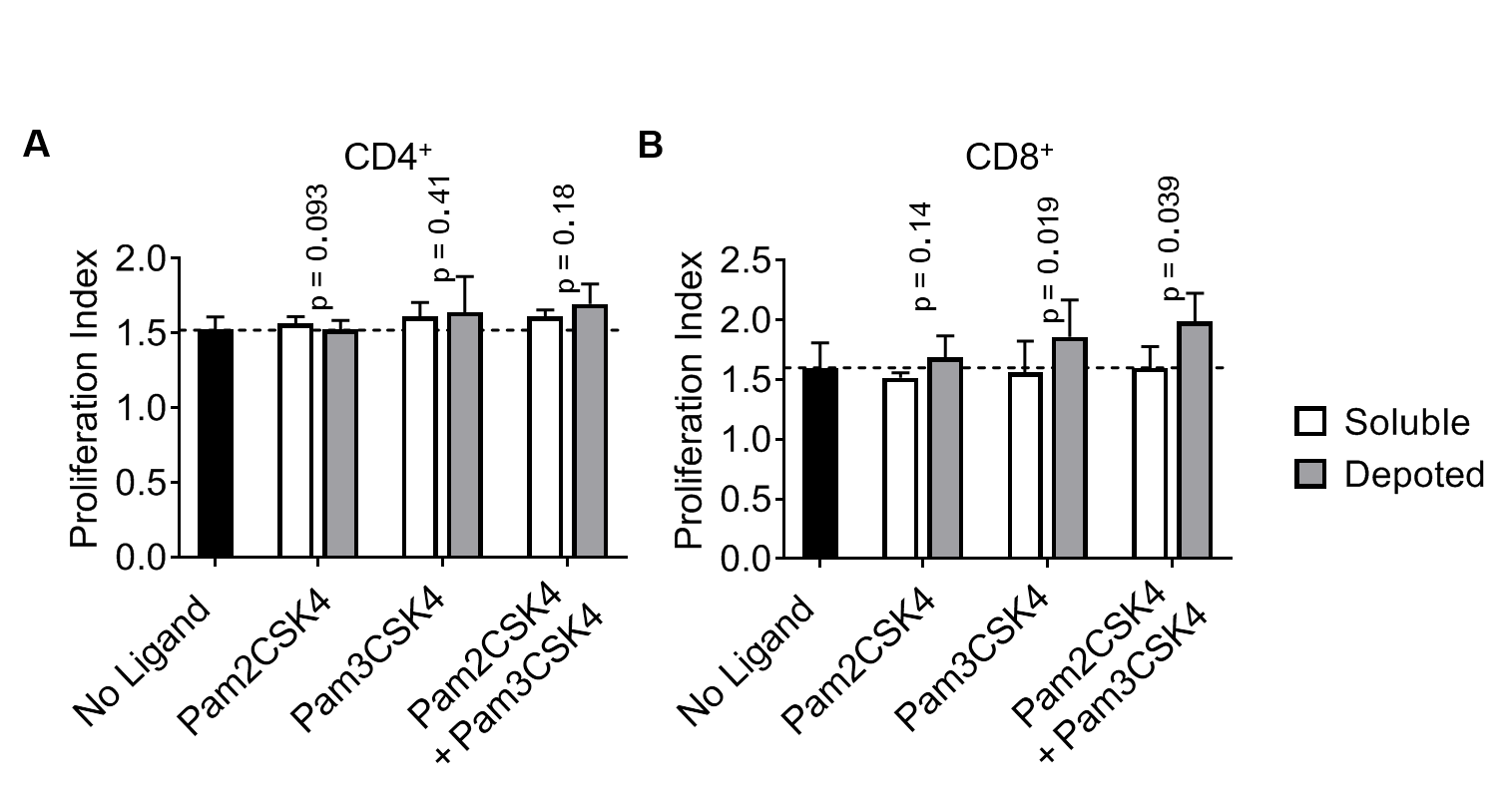

Supplement: Figure S5 — Depoted TLR2 ligands do not enhance proliferation indices of activated CD4+ T cells, but do of CD8+ T cells. Purified polyclonal T cells were stained with 5 μM of carboxyfluorescein succinimidyl ester (CFSE). Different combinations of cell surface ligands (Pam2CSK4 and Pam3CSK4) were either directly added in solution (soluble) or depoted into polyclonal T cells for 1 h and cultured with αCD3/CD28 beads for 3 days. Quantification of proliferation indices of (A) CD4+ and (B) CD8+ T cells in bulk polyclonal T cells as measured by CFSE dilution. Dashed lines represent respective averages (mean) of “No Ligand” controls. p-values by between soluble vs. depoted ligands as determined by one-tailed ratio paired t-test. Data showed m ± s.d. (n = 3 independent samples). [file Image_5.TIF]

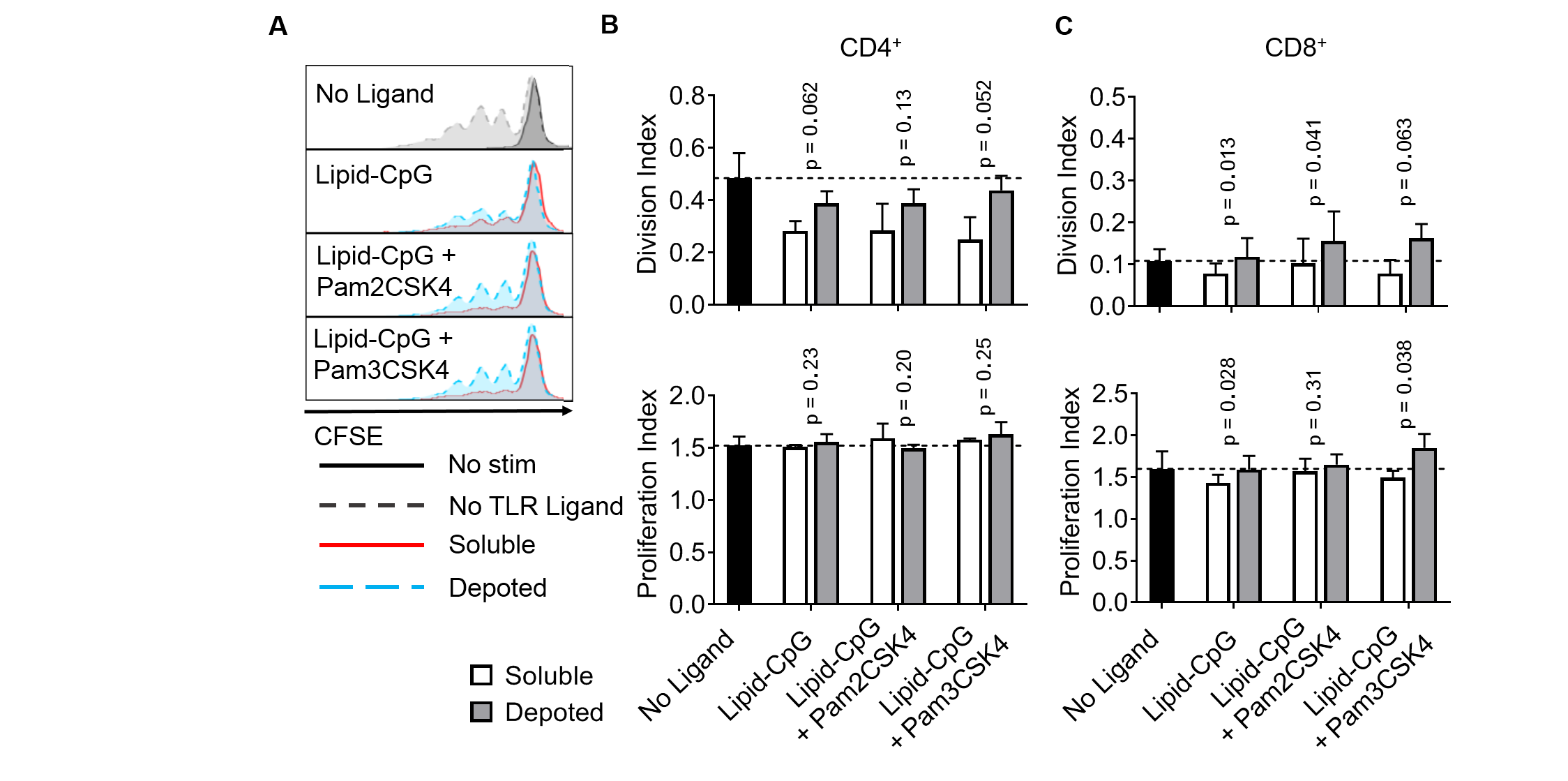

Supplement: Figure S6 — Depoted lipid-TLR9 ligand does not enhance proliferation of activated T cells. Purified polyclonal T cells were stained with 5 μM of carboxyfluorescein succinimidyl ester (CFSE). Different combinations of TLR2 ligands (Pam2CSK4 and Pam3CSK4) and TLR9 ligand (lipid-CpG) were either directly added in bulk solution (soluble) or depoted into polyclonal T cells for 1 h and cultured with αCD3/CD28 beads for 3 days. (A) Representative histograms of CD4+ T-cell proliferation from delivery of lipid-TLR ligand as measured by CFSE dilution. Quantification of division and proliferation indices of (B) CD4+ and (C) CD8+ T cells in bulk polyclonal T cells as measured by CFSE dilution. Dashed lines represent respective averages (mean) of “No Ligand” controls. p-values by between soluble vs. depoted ligands were determined by one-tailed ratio paired t-test. Data showed m ± s.d. (n = 3 independent samples). [file Image_6.TIF]

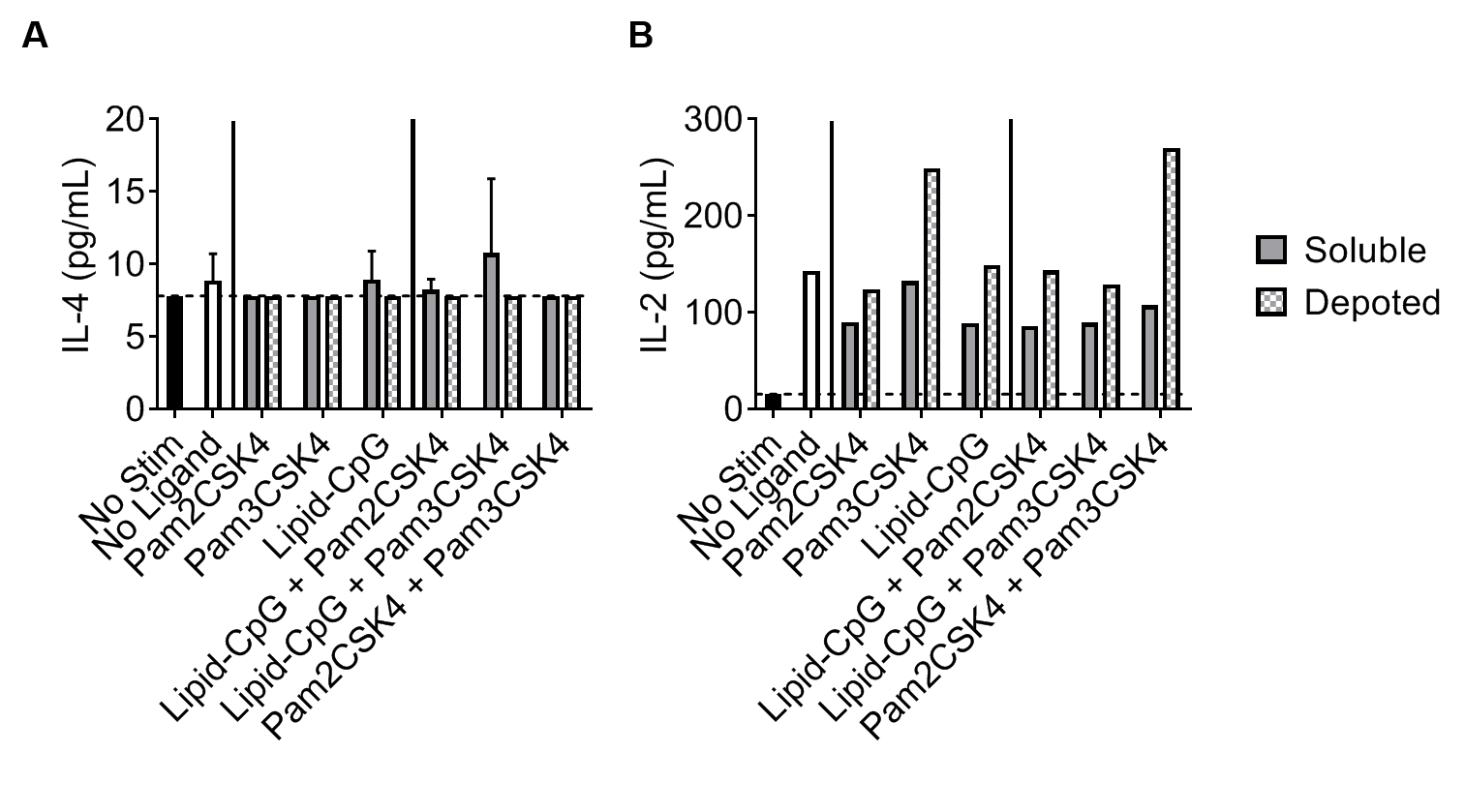

Supplement: Figure S7 — Depoted TLR ligands promote Th1-based T-cell response. Different combinations of TLR2 ligands (Pam2CSK4 and Pam3CSK4) and TLR9 ligand (lipid-CpG) were either directly added in solution (soluble) or depoted into polyclonal T cells for 1 h and cultured with αCD3/CD28 beads for 3 days. Quantification of (A) IL-4 and (B) IL-2 levels in T-cell supernatents as measured by ELISA. Dashed lines represent limit of detection for respective cytokine detection. (n = 1–3 independent samples). [file Image_7.tif]

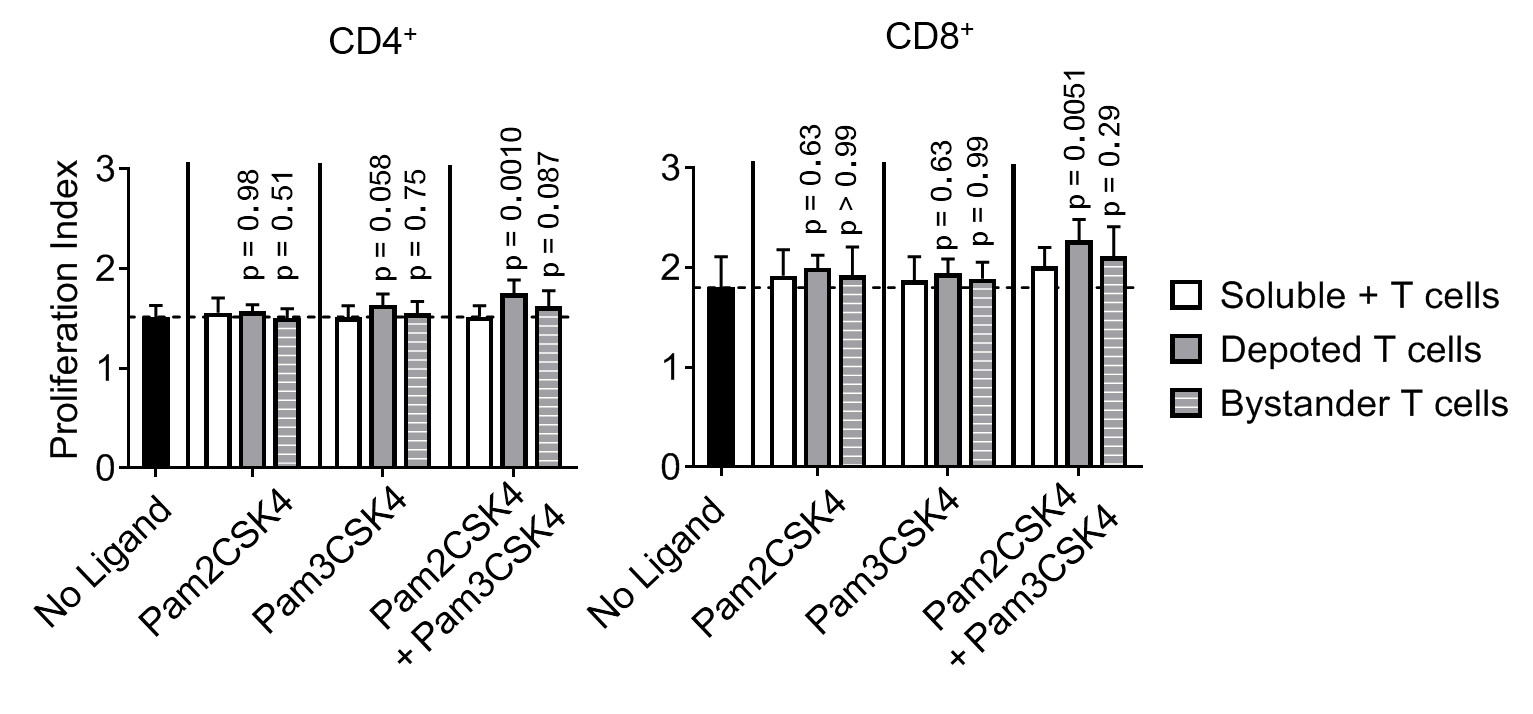

Supplement: Figure S8 — Depoted TLR2 ligands do not enhance cell proliferation indices. Purified polyclonal T cells were stained with 5 μM of carboxyfluorescein succinimidyl ester (CFSE). Different combinations of TLR2 ligands (Pam2CSK4 and Pam3CSK4) were either directly added in bulk solution (soluble) or depoted into stained T cells for 1 h and cultured with non-depoted, stained T cells and αCD3/CD28 beads for 3 days. Quantification of proliferation index of CD4+ and CD8+ T cells in bulk polyclonal T cells as measured by CFSE dilution. Dashed lines represent respective averages (mean) of “No Ligand” controls. p-values within each group is determined by comparing with respective soluble ligand were determined by one-way ANOVA with Sidak's method for multiple comparisons correction. Data showed m ± s.d. (n = 5 independent samples). [file Image_8.TIF]

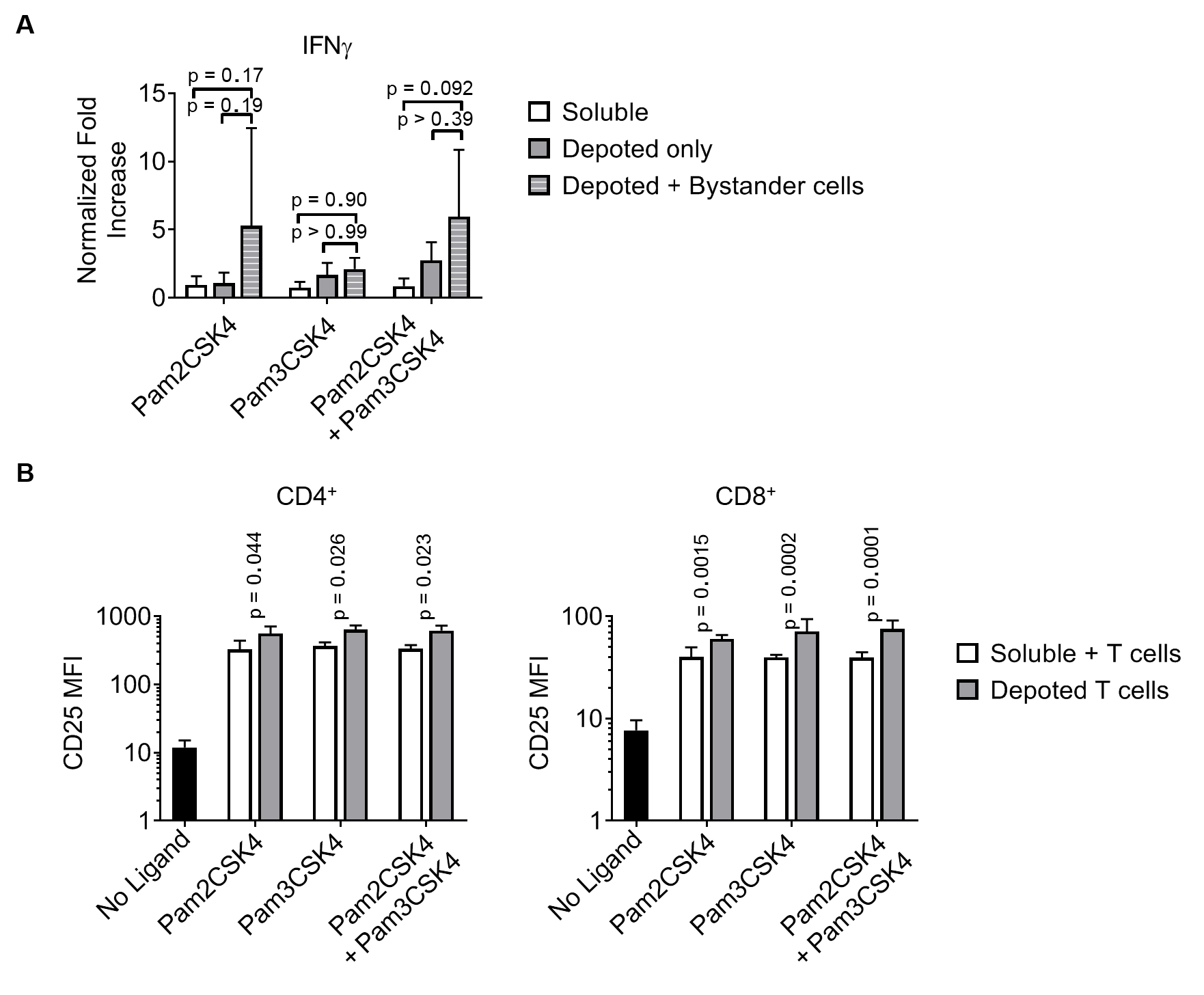

Supplement: Figure S9 — Depoted TLR2 ligands increase CD25 expression on activated T cells. Purified polyclonal T cells were stained with 5 μM of carboxyfluorescein succinimidyl ester (CFSE). Different combinations of TLR2 ligands (Pam2CSK4 and Pam3CSK4) and TLR9 ligand (lipid-CpG) were either directly added in bulk solution (soluble) or depoted into polyclonal T cells and cultured with αCD3/CD28 beads for 3 days. (A) IFNγ from cell supernatants were measured by ELISA on day 2. Concentrations were normalized to αCD3/CD28 bead-stimulated T cells in the absence of TLR2 ligand. p-values between indicated comparisons were determined by two-way ANOVA with Sidak's tests; Data depict m ± s.d. (n = 4 independent samples). (B) CD25 expression as measured by MFI. p-values between corresponding soluble vs. depoted ligands as determined by two-way ANOVA with Sidak's multiple comparisons test; Data showed m ± s.d. (n = 3 independent samples). [file Image_9.TIF]
